# Spencer Wells

**Published:** 1880-09

**Authors:** 


### SPENCER WELLS.

Our readers will feel much interest in learning that last week Mr. Spencer Wells completed his thousandth ovariotomy, and will be gratified to know that the patient is going on well. The results of Mr. Wells' operations, completed or uncompleted, successful or not, have from time to time been with exemplary loyalty and faithfulness, placed before the profession, and the grand statistical outcome of all the thousand ovariotomies he has now performed will, in due course, be brought before the Medical and Chirurgical Society. The record will constitute a singularly great and lasting proof—*monumentum œre perennius*—of Mr. Wells' distinguished and peculiar position among the great surgeons of the nineteenth century. It must have rarely happened, we imagine, that a surgeon has performed any one great operation a thousand times, and certainly, when we remember the opinions held some twenty-five or thirty years ago, by the most eminent surgeons of the day, regarding ovariotomy, the fact that one surgeon has now performed that operation on a thousand patients is one of the most remarkable and striking events in the history of surgeons. The continually increasing success that has attended Mr. Wells' performance of ovariotomy is pretty well known, but the full record of his cases will be expected with great interest.—*Boston Med. and Surg. Journal.*

